# Supplementary material for: Msx1+ stem cells recruited by bioactive tissue engineering graft for bone regeneration
Source: Nat Commun. 2022 Sep 5;13:5211. doi: 10.1038/s41467-022-32868-y (PMC9445030; doi:10.1038/s41467-022-32868-y)
Supplement: Supplementary file 1 — Supplementary Information [file 41467_2022_32868_MOESM1_ESM.pdf]

Msx1^{+} stem cells recruited by bioactive tissue engineering graft for bone regeneration

Xianzhu Zhang #, Wei Jiang#, Chang Xie#, Xinyu Wu, Qian Ren, Fei Wang, Xilin Shen, Yi Hong, Hongwei Wu, Youguo Liao, Yi Zhang, Renjie Liang, Wei Sun, Yuqing Gu, Tao Zhang, Yishan Chen, Wei Wei, Shufang Zhang, Weiguo Zou, Hongwei Ouyang*.

# These authors contributed equally: Xianzhu Zhang, Wei Jiang, Chang Xie.

*Correspondence should be addressed to Hongwei Ouyang. (Email: hwoy@zju.edu.cn)

Supplementary Information

Supplementary Figures and Legends

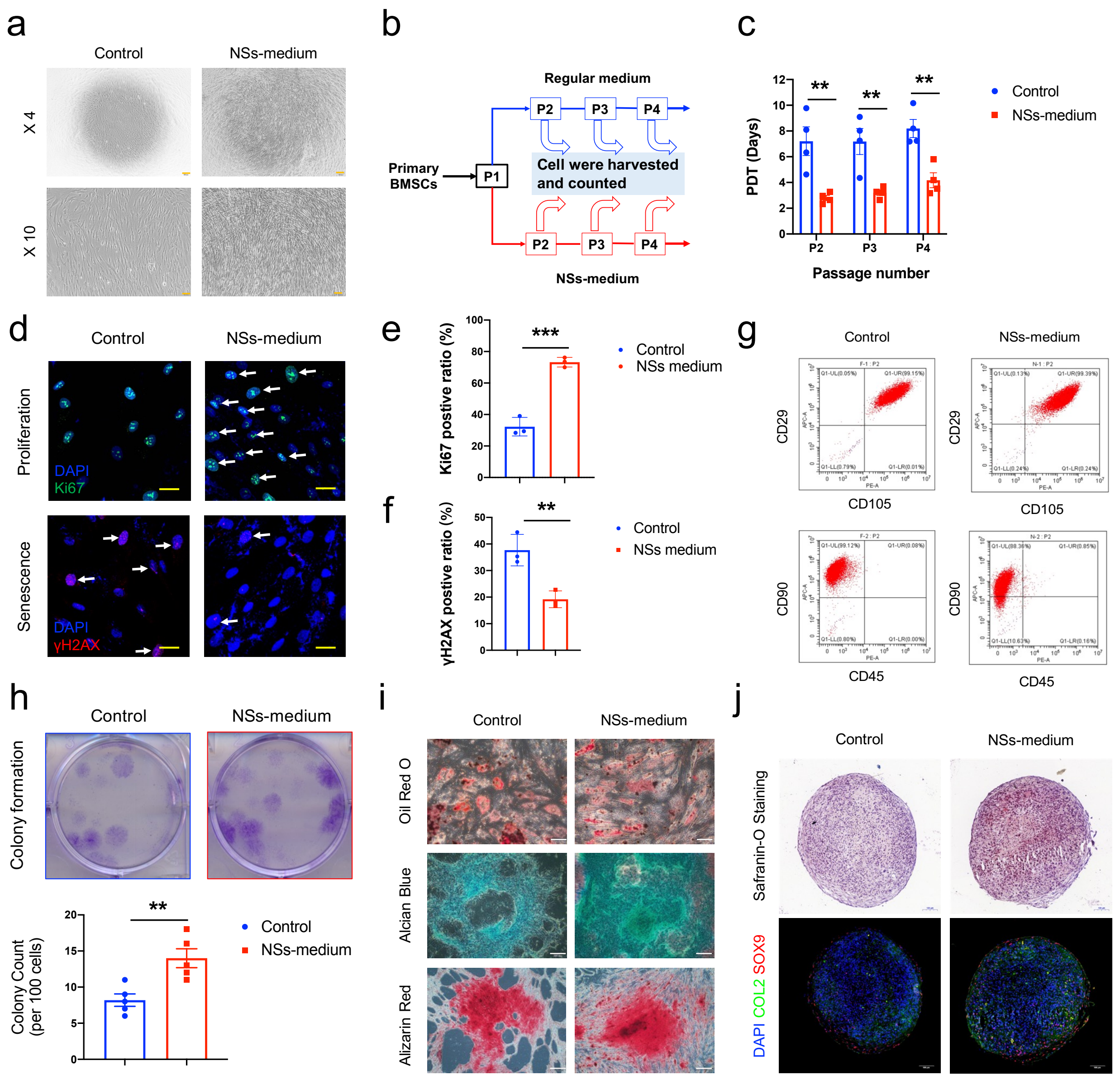

Supplementary Figure 1. Neurotrophic factors maintains the stemness of in vitro cultured MSC. **a**. Bright field image of cell morphology and adhesion after 7 days of NSs treatment when compared with control growth medium (bar=200μm). **b**. Schematic illustration of the serial passage in different culture medium. **c**. Cell population doubling time (PDT) between NSs-contained medium and regular growth medium (n=4 replicates, PDT = Culture Time * log(2)/[log(Final Cell Numbers)–log(Initial Cell Numbers)]), (Exact p-value calculated with unpaired t-test: **p= 0.0079, **p= 0.0086, **p= 0.0045). **d**. Immunofluorescence staining of cell proliferation marker Ki67 (green), bar=30μm, cell senescence marker γh2AX (red), bar=30μm; and **e**. Ki67 positive cell ratio of cultured MSCs, n=3 replicates (Exact p-value calculated with unpaired t-test: ***p= 0.0004) and **f**. γh2AX positive cell ratio of cultured MSCs, n=3 replicates (Exact p-value calculated with unpaired t-test: **p= 0.0088). **g**. Flow cytometry analysis

of the expression of MSCs surface markers (CD105+, CD29+, CD90+, CD45-) after 7 days' culture of NSs-contained medium and growth medium. **h**. Colony formation assay and evaluation (Crystal Violet Staining) showing differences in self-renewal ability (Exact p-value calculated with unpaired t-test: **p= 0.0059, n=5 biologically independent samples). **i**. Tri-lineage differentiation assay and specific staining: adipogenesis (Oil Red Staining), chondrogenesis (Alcian Blue Staining), osteogenesis (Alizarin Red Staining), Scale bar=100μm. **j**. Assessment of the chondrogenic differentiation of MSC by Safranin-O Staining, immunostaining for COL2 and SOX9 in 3D pellet culture model after NSs treatment, Scale bar=100μm. Bone marrow stem cells are isolated from orthopedic individuals with written informed consent (Male, 2 donors, aged 38 and 36). All data represent mean ± SD, and source data are provided as a Source Data file. At least three times of experiments were repeated independently.

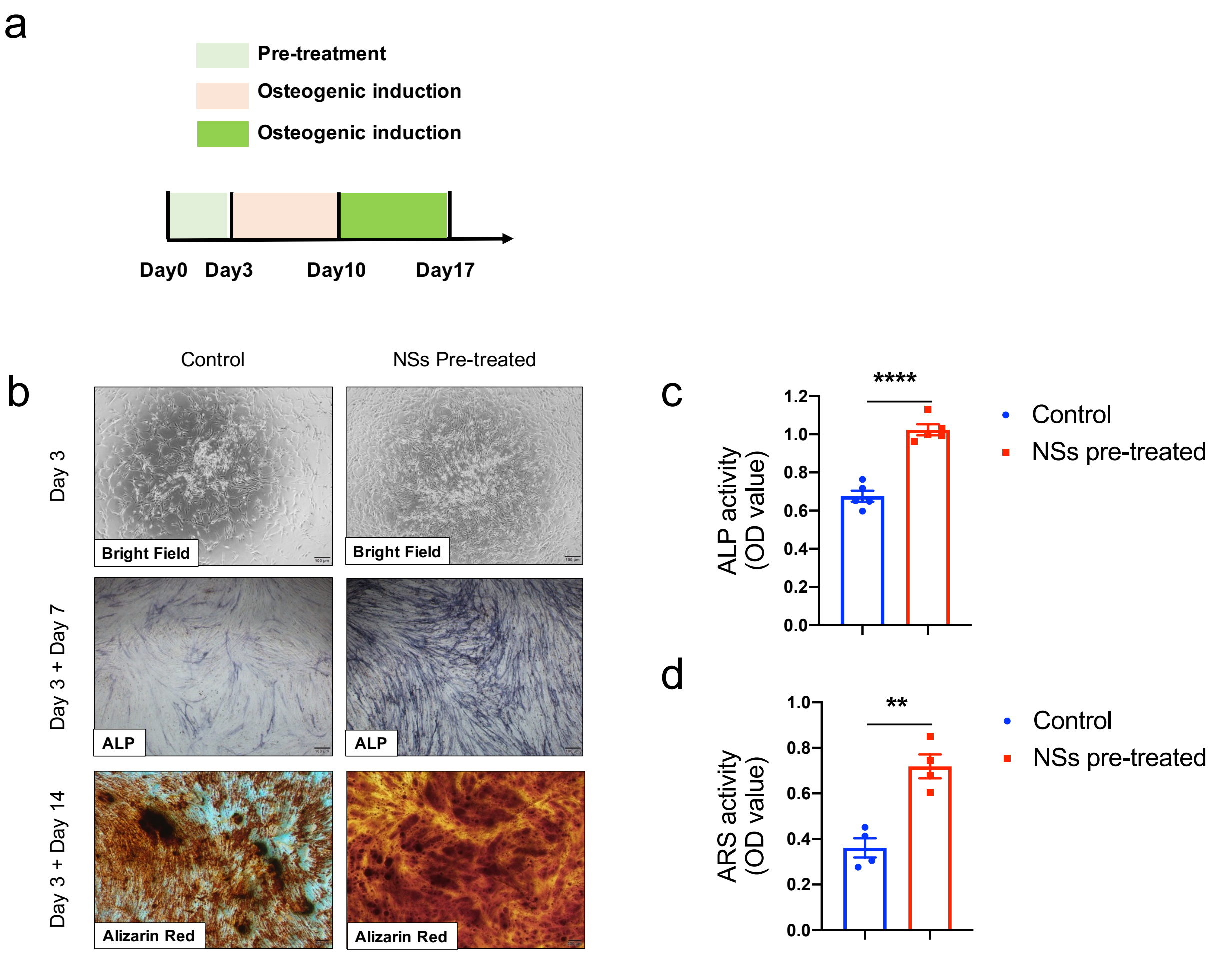

Supplementary Figure 2. Neurotrophic factors pretreatment enhanced the osteogenic potential of MSC. a. Workflow of NSs pre-treatment for 3 days and the subsequent osteogenic induction for 7 days and 14 days. **b**. Bright field image; Alkaline Phosphatase (ALP) staining; Alizarin Red Staining of NSs-pretreated MSCs compared with non-NSs control, bar=100µm. **c** and **d**. The quantitative OD value of ALP activity (c) (Exact p-value calculated with unpaired t-test: **$p < 0.0001$, n=5 biologically independent samples) and ARS activity (d). (Exact p-value calculated with unpaired t-test: **$p = 0.0018$, n=4 biologically independent samples). Bone marrow stem cells are isolated from orthopedic individuals with written informed consent (Male, 2 donors, aged 38 and 36). All data represent mean ± SD, and source data are provided as a Source Data file.

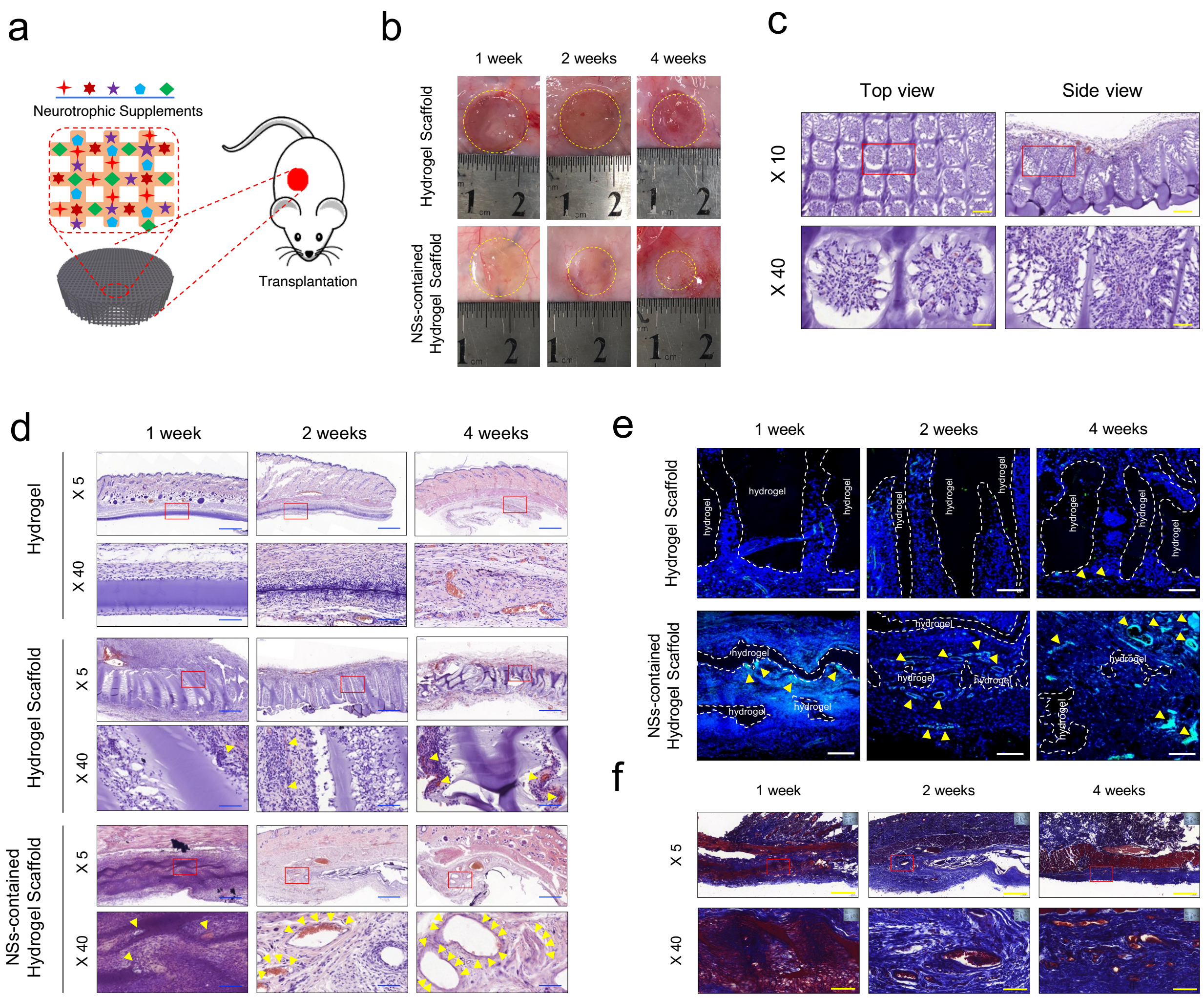

Supplementary Figure 3. 3D printed hydrogel scaffold with NSs promotes in vivo cell compatibility and vascularization. a. Schematic diagram of subcutaneous implantation of the NSs-contained 3D printed hydrogel scaffold system. **b**. Gross view of the vehicle 3D printed hydrogel scaffold and NSs-contained 3D printed hydrogel scaffold at 1, 2, 4 weeks after implantation. **c**. HE staining on paraffin sections of vehicle hydrogel scaffold at 2 weeks after implantation showing *in vivo* cell ingrowth and distribution from top view and side view (bar=200μm; bar=50μm). **d**. HE staining on paraffin sections of hydrogel, hydrogel scaffold, NSs-contained hydrogel scaffold at 1, 2, 4 weeks after implantation (bar=500μm; bar=50μm), the yellow arrowheads indicate red blood cells. **e**. Immunofluorescence staining of CD31 expression on paraffin sections of hydrogel, hydrogel scaffold, NSs-contained hydrogel scaffold at 1, 2, 4 weeks after implantation (bar=50μm), the yellow arrowheads indicate endothelial cells. **f**. Masson' Trichromic staining of paraffin sections in NSs-contained hydrogel scaffold at 1, 2, 4 weeks after implantation (bar=500μm; bar=50μm). At least three times of experiments in sample section staining were repeated independently.

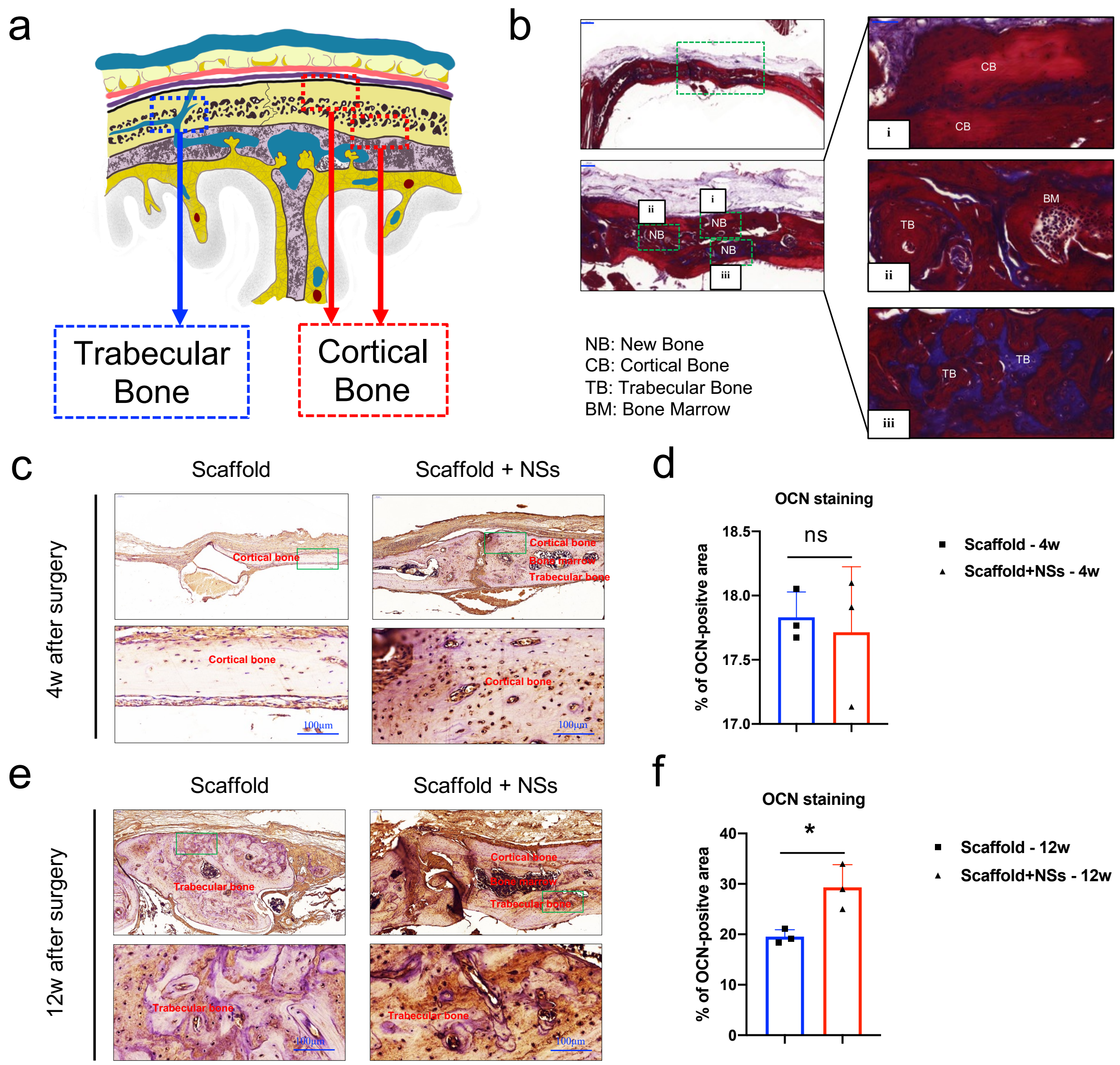

Supplementary Figure 4. 3D printed hydrogel scaffold with NSs promotes full-thickness Calvarial regeneration. a. Schematic of anatomical structure of calvarial bone showing upper and lower layer of cortical bone and middle layer of trabecular bone. **b**. Masson' Trichromic staining of paraffin sections in Scaffold+NSs group at 12 weeks after calvarial defect surgery (bar=500µm; bar=200µm; bar=50µm). **c** and **e**. Immunohistochemical staining of OCN expression in Scaffold, Scaffold+NSs groups at 4 weeks (**c**) and 12 weeks (**e**) after calvarial defect (bar=100µm at high magnification), respectively. **d** and **f**. The quantitative % of OCN-positive staining area in Scaffold, Scaffold+NSs groups at 4 weeks (**d**) (ns, not significant) and 12 weeks (**f**) (Exact p-value calculated with unpaired t-test: 0.0225, n=3 biologically independent rats) after calvarial defect, respectively. All data represent mean ± SD, and source data are provided as a Source Data file. At least three times of experiments in section staining were repeated independently.

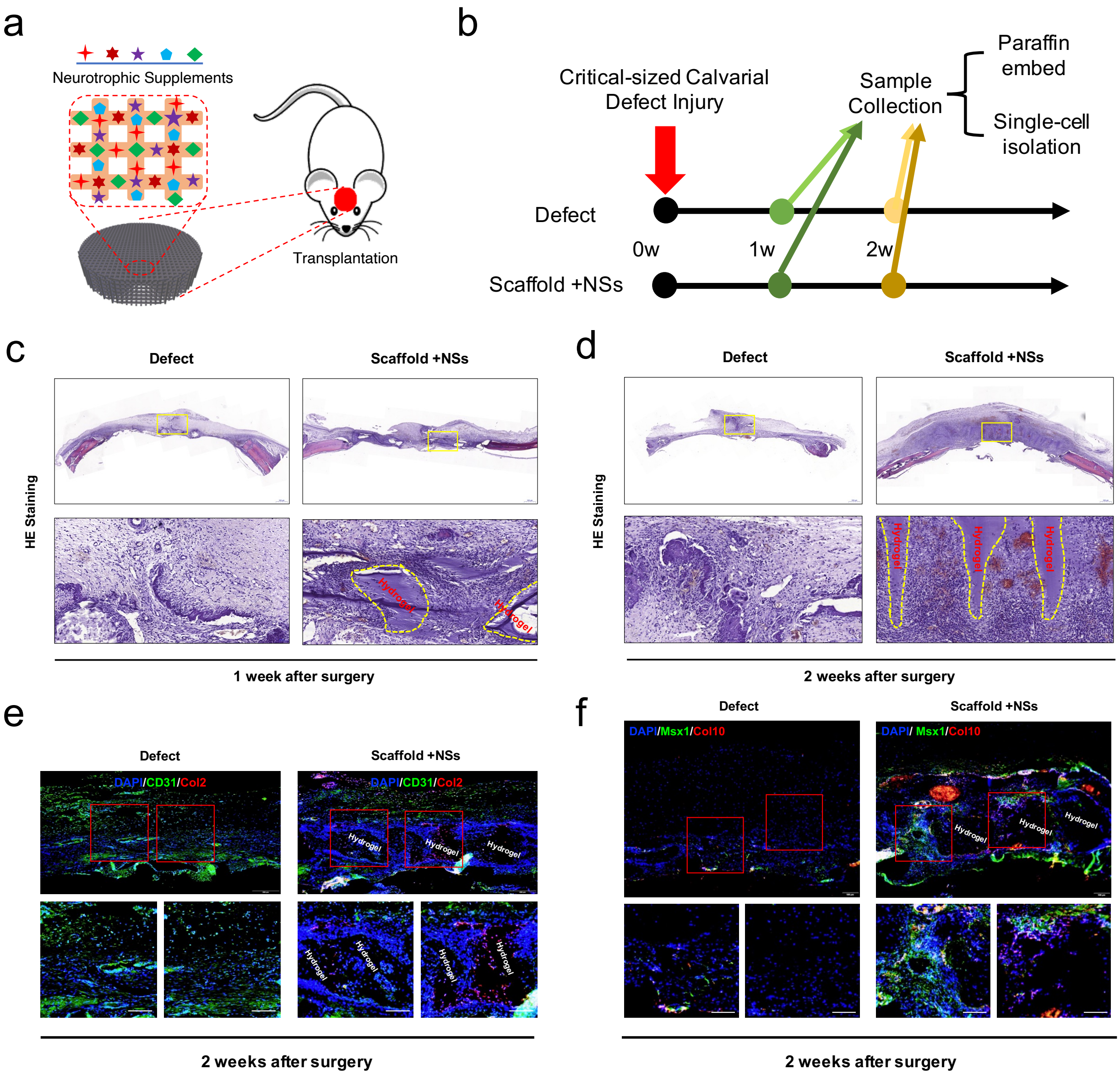

Supplementary Figure 5. 3D printed hydrogel scaffold with NSs promotes in situ cell expansion. **a**. Illustration of the transplantation of NSs-loaded 3D printing gel scaffold in calvarial defect model. **b**. The schematic of the flow chart of sample collection for scRNA-Seq analysis and histological assessments at 1week and 2 weeks after defect surgery, n=6 rats per group and per time point. **c** and **d.** HE staining of paraffin sections in Defect and Scaffold + NSs group at 1 week and 2 weeks after calvarial defect surgery, respectively (bar=500μm; bar=50μm). **e** and **f.** Co-immunostaining of (**e**) CD31 and Col2 expression and (**f**) Msx1 and Col10 expression on paraffin sections in Defect and Scaffold + NSs group at 2 weeks after defect surgery (bar=200μm at low magnification and bar=100μm at high magnification). At least three times of experiments in section staining were repeated independently.

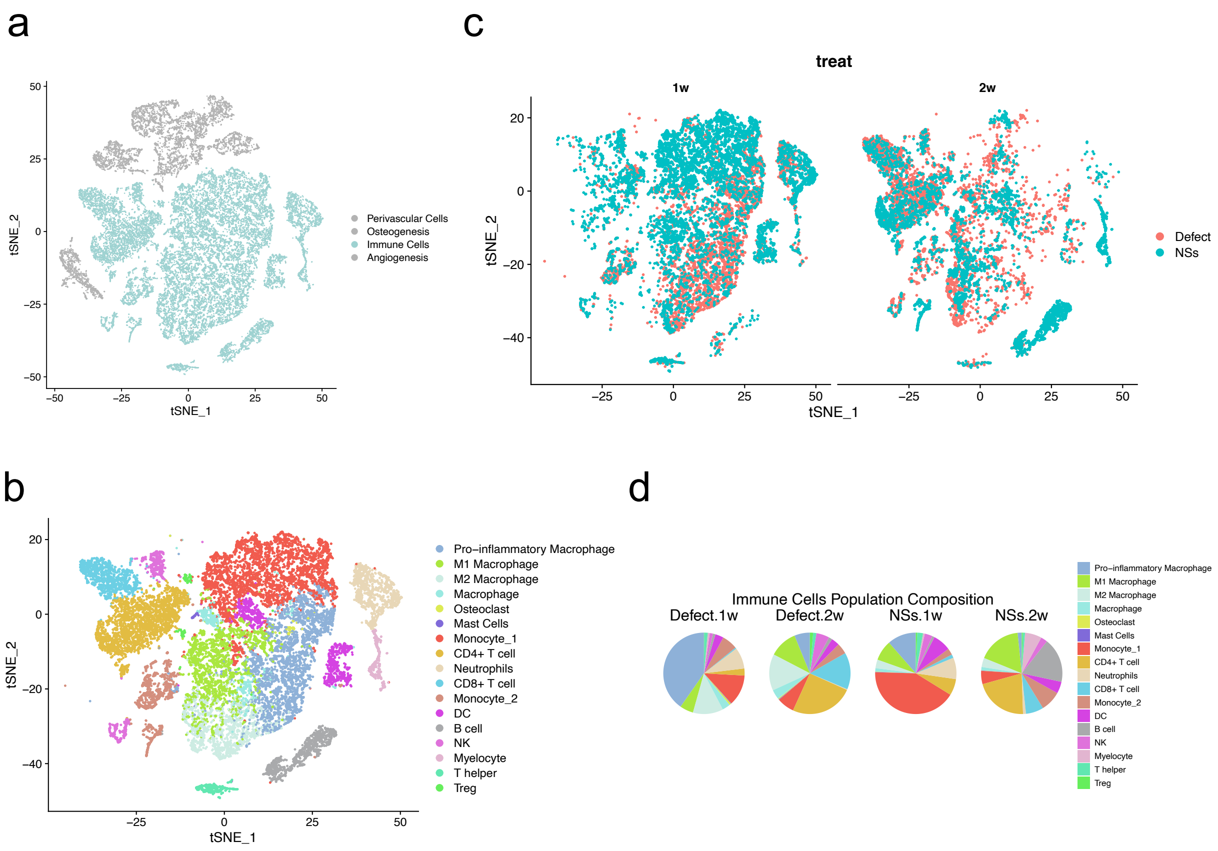

Supplementary Figure 6. Immune cells are widely modulated by *in situ* culture system with NSs. **a**. tSNE plot of the immune-lineage cell population among all the four different cell populations . **b**. tSNE plot showing the distribution of immune-lineage subpopulations. **c**. tSNE plot of differential distribution of immune-lineage cells regulated between Defect and NSs group from different time points. **d**. Relative proportions of cell subpopulations from Defect and NSs group in each timepoints.

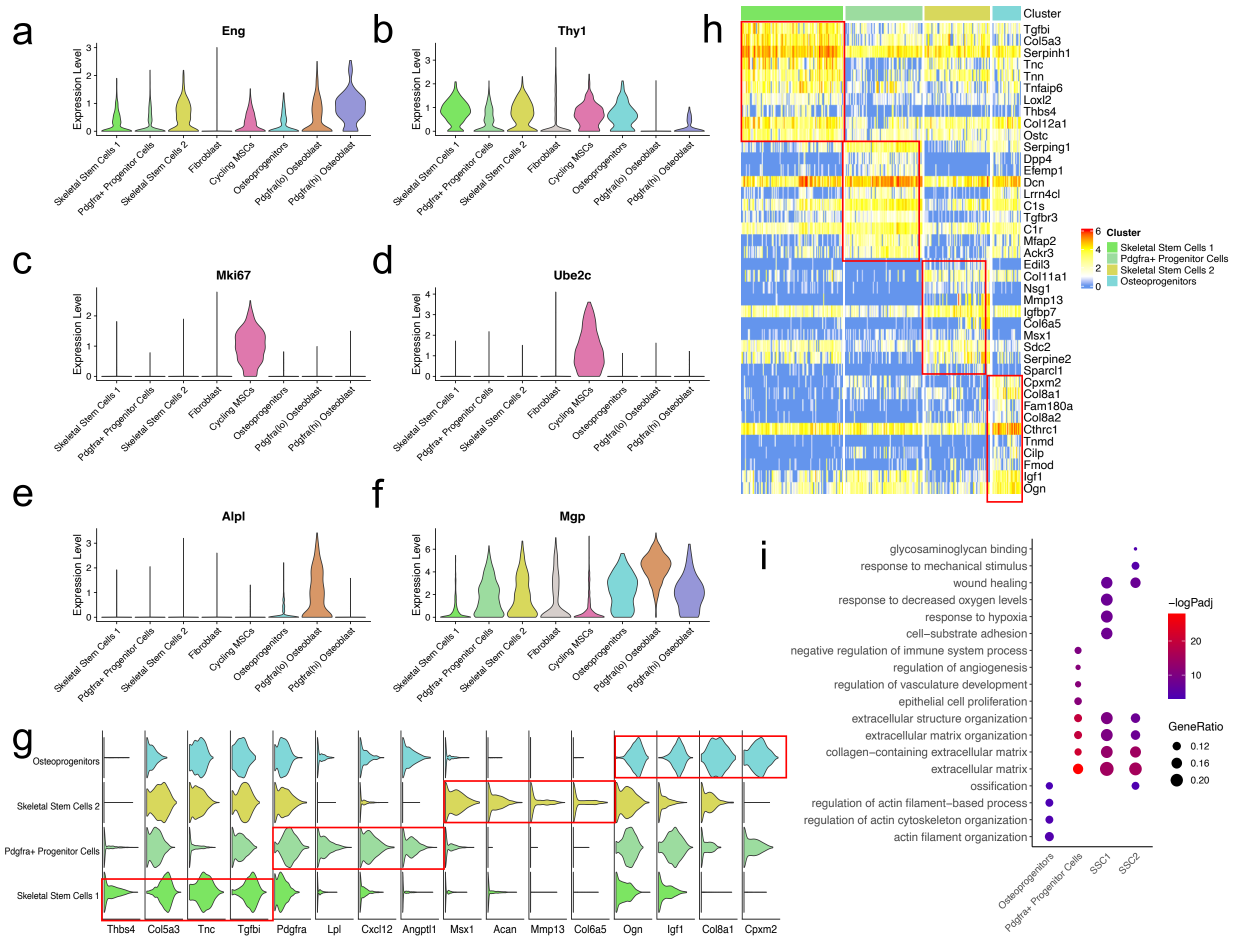

Supplementary Figure 7. scRNA Seq analysis reveals four skeletal stem/progenitor cells in osteo-lineage cell population. a-f. Violin plots showing specific gene expression levels in different subsets (Fibroblast, negative for Eng and Thy1 expression; Cycling MSCs, Mki67 and Ube2c expression; Osteoblasts, Alpl and Mgp expression). **g**. Violin plot shows expression of specific marker genes in four skeletal stem/progenitor cell subsets. **h**. Heatmap showing expression of Top 10 cell markers of four skeletal stem/progenitor cell subsets. Marker genes were selected according to p value and fold change (Wilcoxon Rank Sum test, Top 10). Marker genes are provided in source data. **i**. Dot plots showing the GO enrichment in four skeletal stem/progenitor cell subsets. Dot size represented the proportion of genes enriched to GO and color bar represented the p adjusted value (performed by R package clusterProfiler, Benjamini-Hochberg).

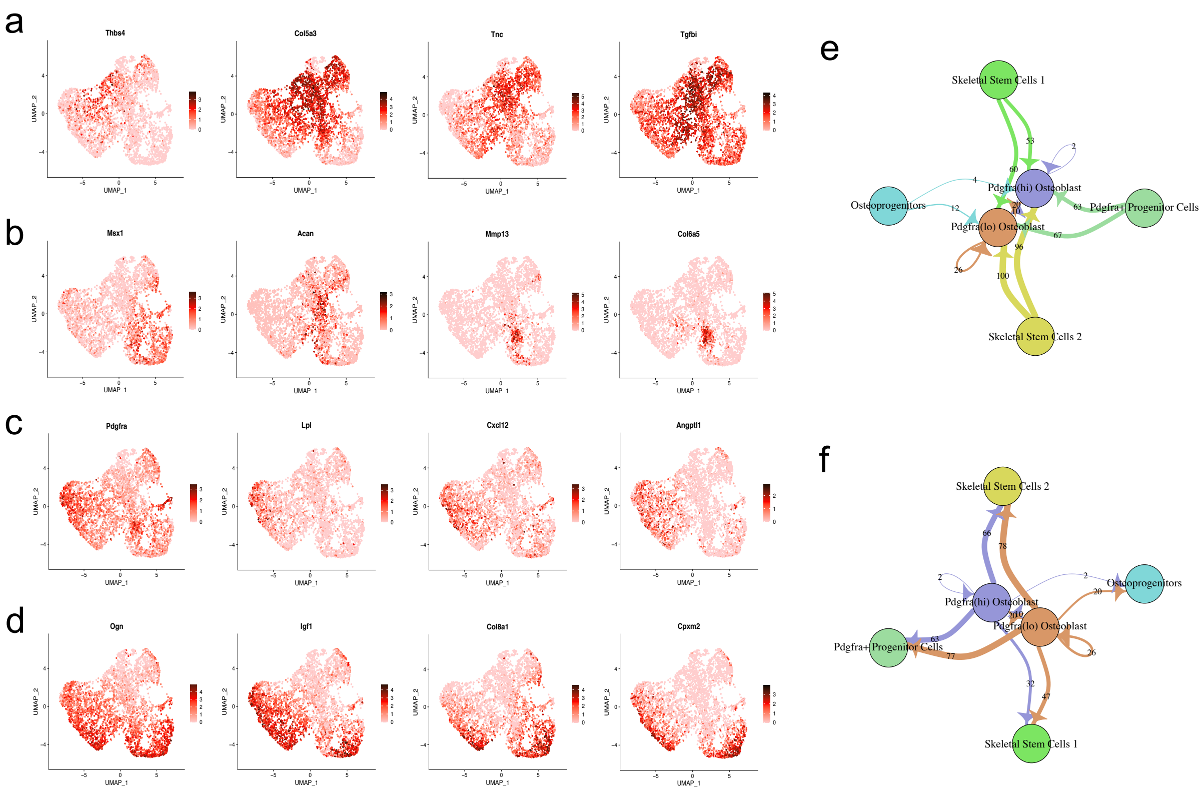

Supplementary Figure 8. Four skeletal stem/progenitor cells showed close distribution in UMAP. a-d. Feature plots showing the expression of specific marker genes in four skeletal stem/progenitor cell subsets. **e** and **f**. Network plot showing the number of ligand-receptor interactions detected between each the four skeletal stem/progenitor cell subsets and osteoblasts subsets.

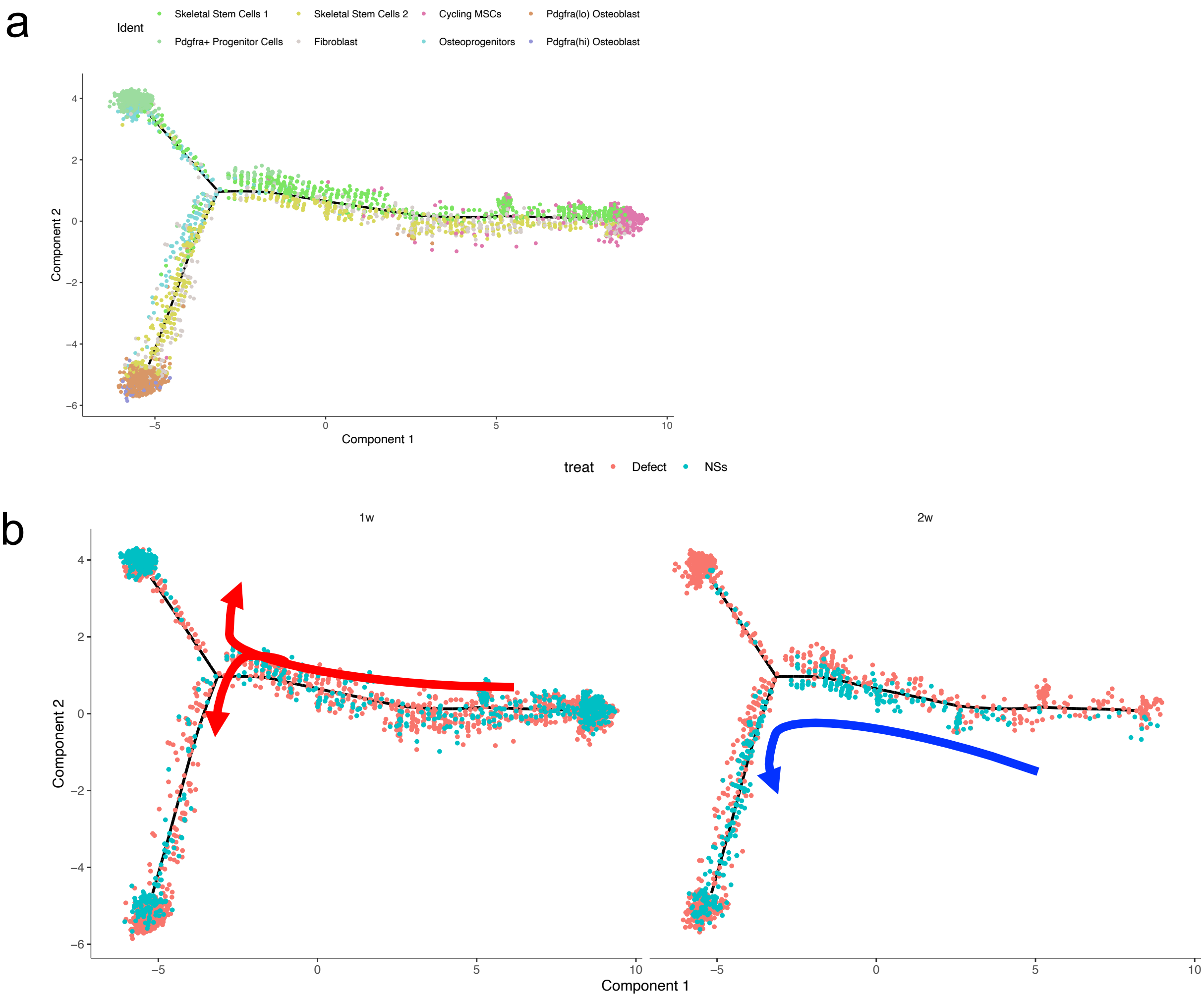

Supplementary Figure 9. Pseudotime ananlysis of two different trajectories in SSCs differentiation. a. Differentiation trajectory of from cycling MSCs forward to SSC1 and SSC2 lineage cells predicted by monocle 2. **b**. Distribution of cells on both the differentiation trajectories at 1week and 2weeks after surgery, showing distinct differentiation patterns dominated by Defect and NSs, respectively.

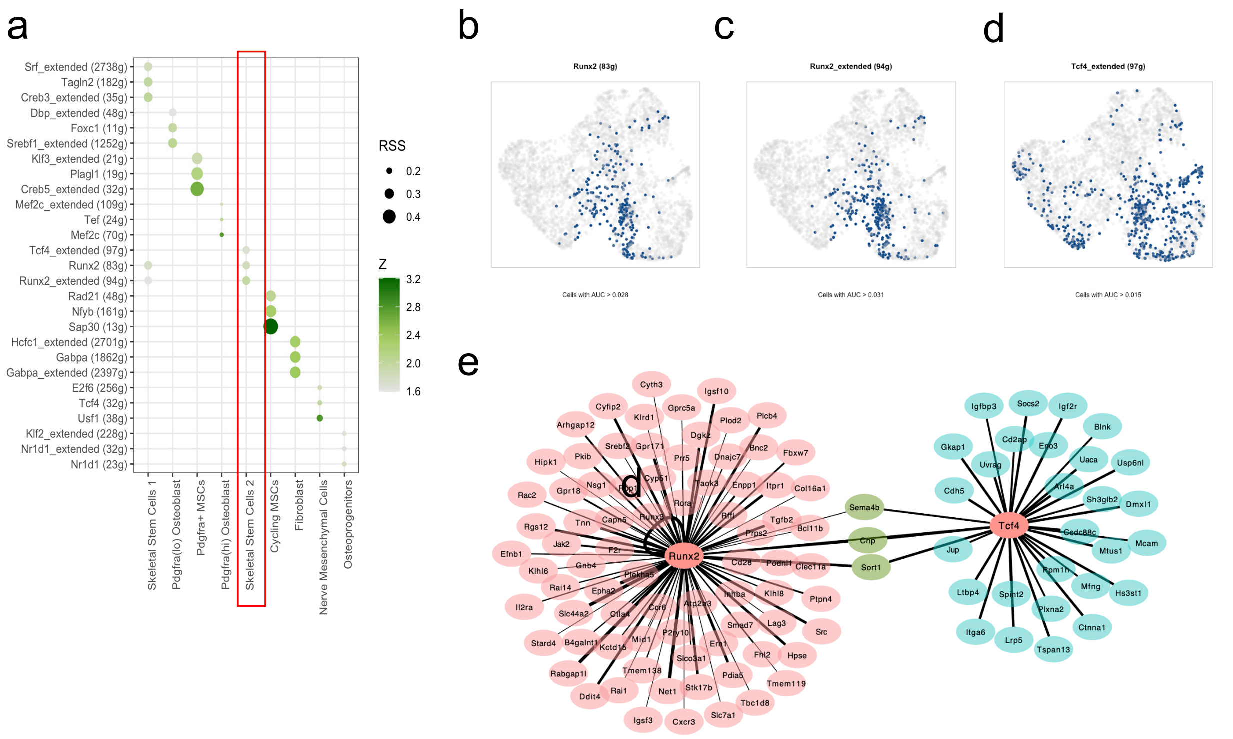

Supplementary Figure 10. Regulon analysis of top transcription factors in SSC2. a. Dotplot shows the cell-type specific regulons with top Regulon Specificity Score (RSS) and their average expression (Z) in the cell subtype. **b-d**. Binarized regulon activity of the key regulons (Runx2 and Tcf4) visualized in UMAP. **e**. Regulation network of Runx2 and Tcf4 with overlap target genes. The line width indicated the Normalized Enrichment Score (NES) inferred by RcisTarget of regulon-target pair.

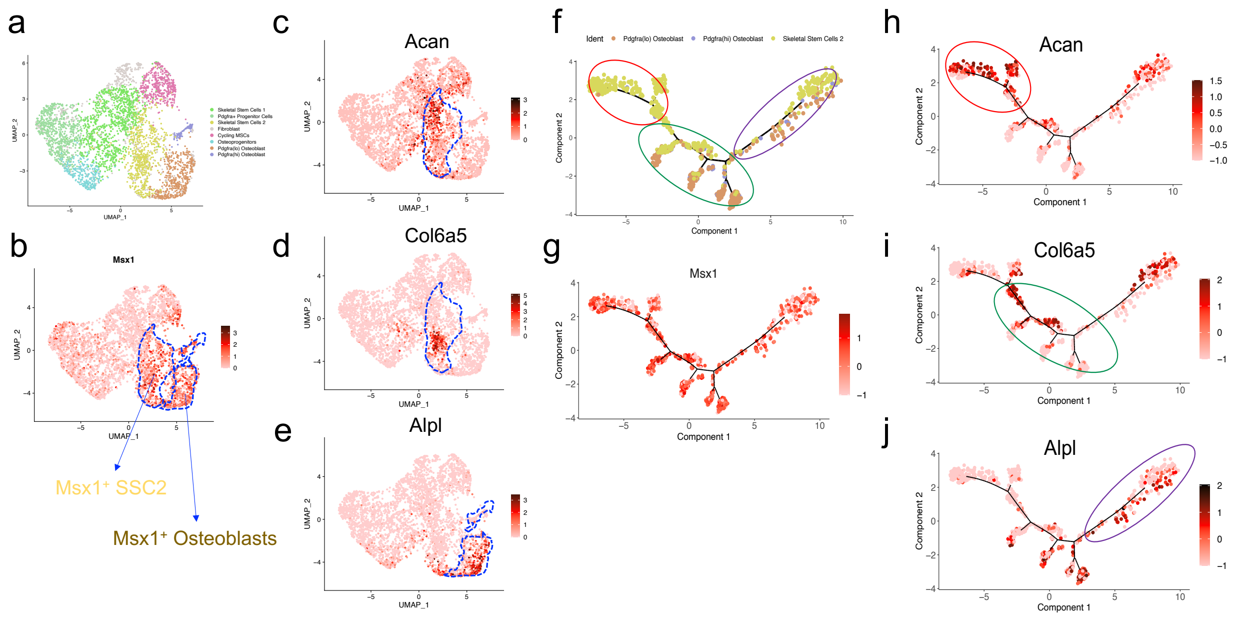

Supplementary Figure 11. Pseudotime analysis models the relationship between SSC2 and Osteoblasts. a. Visualization of eight osteo-lineage cell sub-clusters with UMAP plot. **b**. UMAP plot shows the expression of Msx1, highlighting Msx1+ SSC2 and Msx1+ Osteoblasts. **c-e**. UMAP plot shows the expression of specific genes (Acan, Col6a5, Alpl). **f**. Trajectory of differentiation composed of Msx1+ SSC2 and Msx1+ Osteoblasts that predicted by monocle 2 analysis. **g-j**. Pan-expression of Msx1 and region-specific expression of genes (Acan, Col6a5, Alpl) visualized on continuous trajectory.

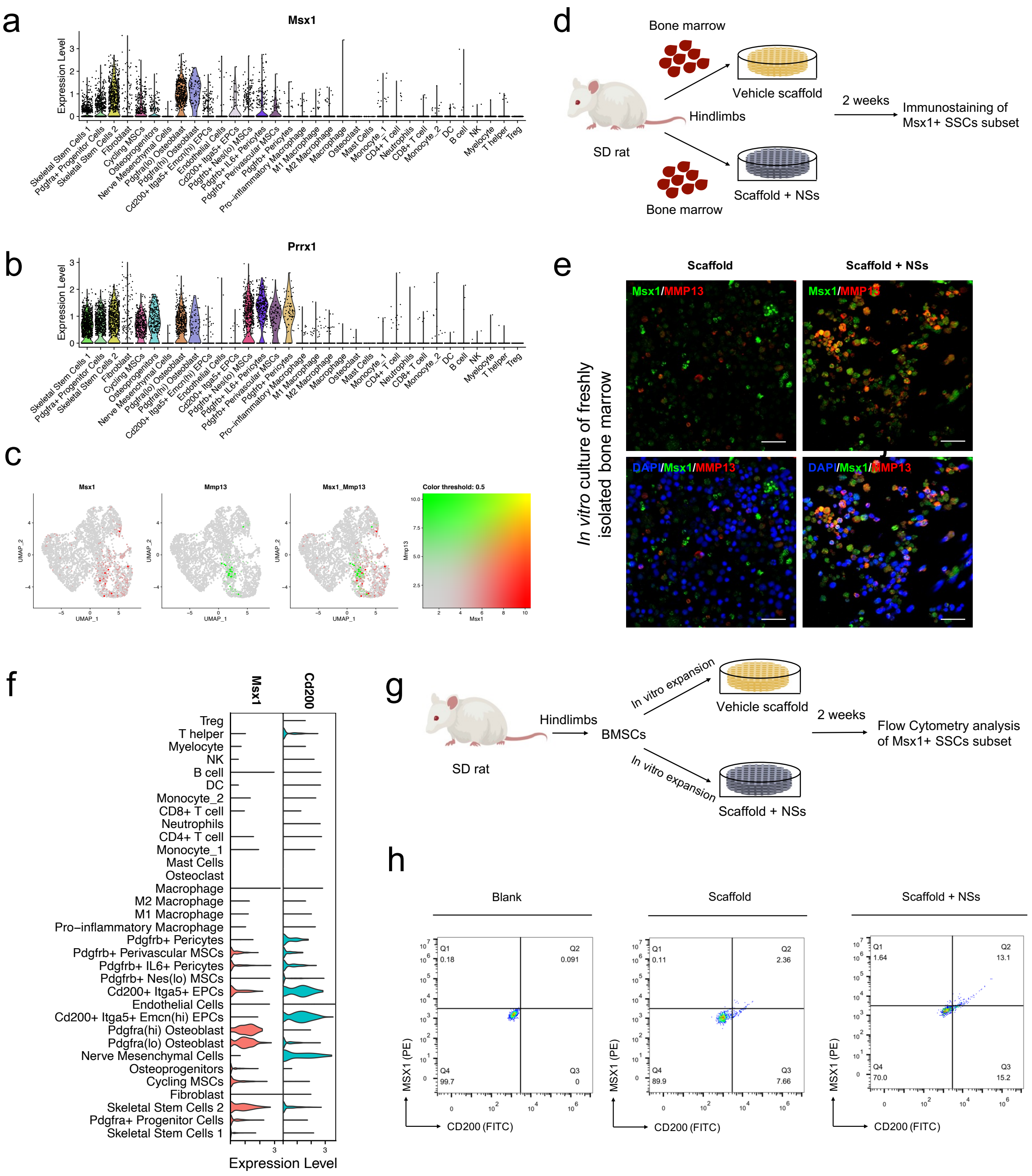

Supplementary Figure 12. NSs-loaded hydrogel culture system promotes the *in vitro* expansion of Msx1+ SSC2 subset. a, b. Violin plots showing Msx1 and Mmp13 expression levels in all cell subsets of regenerated skull tissue, respectively. **c**. Visualization of expression and co-expression of Msx1 and Mmp13 in UMAP plot. **d**. Experimental design for the *in vitro* expansion of freshly isolated rat bone marrow. **e**. Co-immunostaining of Msx1 and Mmp13 expression of primary bone marrow tissue after expansion for 2 weeks on the culture system of NSs-loaded 3D printed hydrogel scaffold

(bar=30μm). **f**. Violin plots showing Msx1 and CD200 expression levels in all cell subsets of regenerated skull tissue, respectively. **g**. Experimental design for the *in vitro* expansion and Flow cytometry analysis of rat BMSCs. **h**. Flow cytometry analysis of rat BMSCs. Left panel: Blank; Middle panel: analysis of Msx1^{+} SSC2 for MSX1 expression; Right panel: analysis of Msx1^{+} SSC2 for CD200 expression. At least three times of experiments were repeated independently.

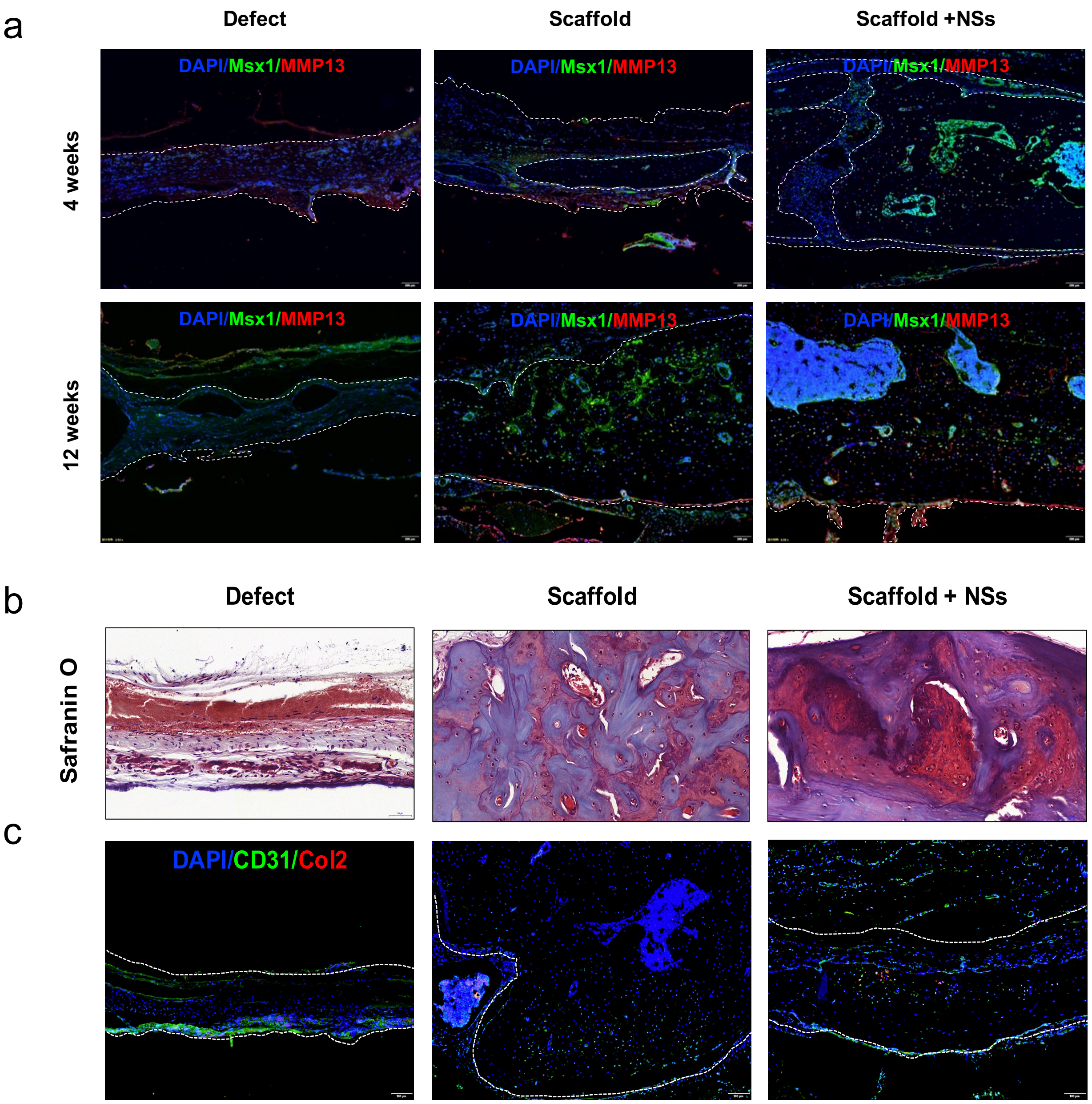

Supplementary Figure 13. NSs promotes calvarial regeneration with endochondral bone formation. a. Co-immunofluorescent staining of Msx1 and Mmp13 expression in Defect, 3D Scaffold, Scaffold + NSs groups at 1 and 3 month after rat calvarial defect (bar=200μm), respectively. **b**. Safranin-O staining of paraffin sections in Defect, 3D Scaffold, Scaffold + NSs groups at 3 month after rat calvarial defect (bar=50μm). **c**. Co-immunofluorescent staining of CD31 and Col2 expression in Defect, 3D Scaffold, Scaffold + NSs groups at 3 month after calvarial defect (bar=200μm). At least three times of experiments in sample section staining were repeated independently.

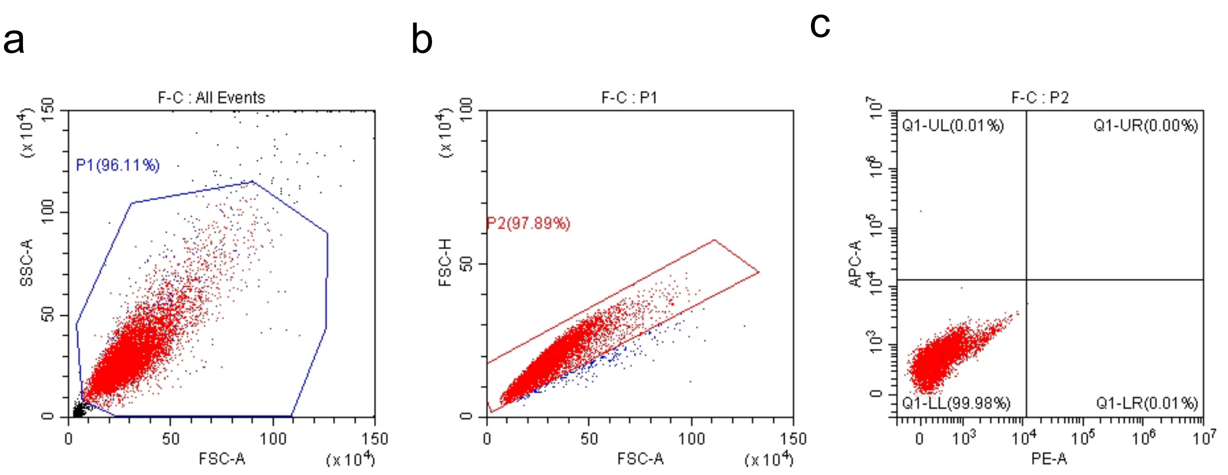

Supplementary Figure 14. Gating strategy used for flow cytometry analysis of expanded cells. All cells were first gated on FSC/SSC, according to cell size and granularity, using unstained cells as a negative control to decide the background values and boundaries.
